# Mechanistic Insights into MoS_2_ Functionalization via Thiol Groups of 4‑Aminothiophenol

**DOI:** 10.1021/acs.langmuir.6c01436

**Published:** 2026-06-29

**Authors:** Jon Azpeitia, Jesús Ignacio Mendieta-Moreno, Laia León-Boigues, Mar García-Hernández, José Ángel Martín-Gago, Carmen Munuera, Irene Palacio

**Affiliations:** Instituto de Ciencia de Materiales de Madrid, 69570 CSIC, Sor Juana Inés de la Cruz 3, Cantoblanco, Madrid 28049, Spain

## Abstract

Functionalization of transition metal dichalcogenides is a key process for future technological applications. To date, various routes, primarily chemical, have been proposed for efficient functionalization with thiol moieties. Previous functionalization protocols have generally taken advantage of intrinsic defects typically present on the surface. However, the specific nature of the molecule–substrate bond, covalent or noncovalent, has always remained controversial. In this work, we present a study of the functionalization of MoS_2_ by physical vapor deposition in ultrahigh vacuum conditions of the 4-aminothiophenol molecule. We have investigated its interaction with the intrinsic sulfur vacancies present in MoS_2_ and with additional vacancies generated by ion bombardment. We observe that the molecule is found in planar configuration on the surface and the thiol moiety remains intact. Atomic force microscopy measurements show high diffusion of molecules through the surface, along with a strong interaction between the thiol moieties and the tip itself. Interestingly, this interaction leads to a contrast inversion in the acquired AFM images, which is a key observation that leads to consider that molecules are not covalently bonded. Density functional theory calculations rule out thiol dehydrogenation, attributing the observed contrast inversion to molecular tip functionalization. These findings provide compelling evidence of a nonspecific physisorption interaction, confirming the absence of covalent bonding between the molecule and the surface.

## Graphical abstract

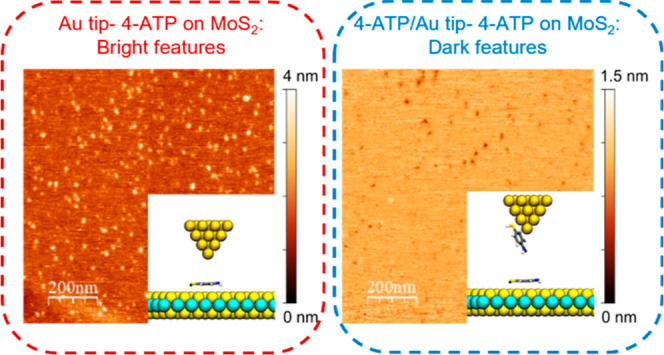

## Introduction

Among 2D materials, the relevance of transition metal dichalcogenides (TMDs) has increased dramatically in the past few years, particularly in the case of MoS_2_. Their intrinsic properties in the nanoscale have opened new paths for applications in photonics and optoelectronics,^1^ catalysis,^2^ biosensing,^3^ or energy storage^4^ among others.^5^ In addition, those intrinsic properties can be tailored by functionalizing the surface through different strategies. Functionalization can be performed through chemical or physical approaches. Chemical functionalization involves the incorporation of molecules into the surface under study using chemical methods, where the functionalizing species are diluted in different solvents,^6^ whereas physical functionalization is based on the incorporation of functionalizing agents commonly by vacuum deposition.^7^ Depending on the nature of the interaction between the incorporated species and the surface, functionalization can be noncovalent or covalent. Both have demonstrated successful functionalization of different materials.^8–10^ Noncovalent functionalization involves physisorption through weak interactions such as electrostatic forces or π–π stacking. In this case, the structure of the surface is preserved but the stability of the molecules on the surface and the reproducibility of the functionalization process can be compromised. Covalent functionalization involves a chemical bond between the molecule and the surface, which leads to a more stable functionalization, but requires high process control and can sometimes damage the material. Due to the high chemical stability of TMDs and in particular MoS_2_, many efforts have been made not only in the development of functionalization protocols, but also in the implementation of protocols to modify or activate its surface, thus obtaining a more effective functionalization, such as interface engineering^11^ or defect engineering.^12^ The chemical and structural characteristics of MoS_2_ also require the use of specific molecules for its covalent functionalization. Diazonium salts and thiol-containing molecules are among the most commonly used functionalizing species. In the case of diazonium salts, covalent bonding occurs through the creation of radicals that interact with the surface, regardless of its reactivity,^13–17^ while thiol-containing molecules require reactive sites, such as sulfur vacancies that may lead to the dehydrogenation of the thiol moiety.^18–24^ However, in the case of the use of thiolated molecules, the unequivocal demonstration of covalent functionalization remains a point of controversy in the scientific community. In previous studies conducted using wet chemistry methods, the nature of the covalent bond has not been directly proven. For instance, McDonald et al. present the covalent functionalization with cysteine of 2H-MoS_2_ through the formation of dative bonds with Mo-atoms at the sulfur vacancies, nonetheless suggesting that a physisorption process cannot be ruled out.^25^

In this work, we aim to demonstrate that the commonly assumed mechanism—namely that thiols dehydrogenate at sulfur vacancies in MoS_2_ and form covalent bonds due to the enhanced reactivity of these sites—does not occur under our experimental conditions. Our results show that this binding does not take place at sulfur vacancies, nor does it occur on pristine MoS_2_ surfaces. For this purpose, we have employed the 4-aminothiophenol (4-ATP) molecule, which contains a thiol moiety, as a model candidate for MoS_2_ functionalization in ultrahigh vacuum (UHV) environment via direct evaporation at room temperature. This protocol, where no solvents are involved, ensures a clean functionalization process. Furthermore, it is important to note that traditional wet chemistry methods have not yet provided definitive evidence of covalent functionalization in this specific system, further justifying our solvent-free approach.^25^ We have studied the pristine surface and the molecule-decorated surface by scanning tunneling microscopy (STM) and atomic force microscopy (AFM) to evaluate the morphology of both, the pristine and functionalized surfaces and quantify the density of defects together with the density of molecules at different doses. Furthermore, we have performed experiments on a surface with artificial point defects generated by Ar^+^ sputtering, in order to unequivocally elucidate the role of sulfur vacancies. For both types of samples, we found that dehydrogenation of the thiol moiety does not take place and the molecule remains intact in a planar geometry on the surface. Therefore, and contrary to previous reports, these defects do not play an active role as reaction sites for the covalent binding of molecules to the surface, as evidenced by both the high diffusion of molecules across the surface and their interaction with a gold AFM probe precisely due to the high affinity of thiols with gold. Furthermore, DFT calculations conclude that deprotonation of the thiol group does not occur and prove that the strong interaction between the AFM probe and the 4-ATP molecules results in a change in contrast in the acquired images. The behavior of thiol-containing molecules may differ depending on the backbone of the molecule, presenting different adsorption energies and geometries.^26^ In the present work, the experimental findings, combined with theoretical calculations, point toward a nonspecific adsorption of these particular molecules on the surface.

## Materials and Methods

After exfoliation in ambient conditions to ensure a clean surface, synthetic 2H-MoS_2_ single crystal samples (2D semiconductors) were introduced in UHV conditions (base pressure 1 × 10^–10^ mbar). STM images were acquired with a flow-cryostat STREAM STM (Scienta Omicron GmbH) at 77 K. A commercial atomic force microscopy (AFM) from Nanotec Electrónica S. L., operating in ambient conditions was employed to obtain topographic images. Measurements were performed in dynamic mode by exciting the cantilever at its resonance frequency, using the oscillation amplitude as the feedback signal and with additional phase-locked loop (PLL) feedback enabled. Silicon tips from Nanosensors (PPP-FMR and SSS-FMR) and AuNPs-modified tips from NextTip (NT-TP-HRES-75) were employed. AuNPs-modified probes were selected instead of conventional Au-coated tips in order to preserve the high spatial resolution required for molecular identification. The attachment of 2–3 nm Au nanoparticles minimally alters the apex geometry and increases the aspect ratio, thus maintaining high resolution while enabling gold–thiol interactions.^27^ STM and AFM images were processed and analyzed with WSxM program from Nanotec.^28^ 4-ATP molecules, in powder form (97% purity, Sigma-Aldrich), due to their high vapor pressure, were directly evaporated at RT from a glass crucible, with the sample at RT, controlling the dose by controlling the pressure (7 × 10^–7^ mbar) monitored by a Bayard-Alpert pressure gauge, and varying the exposure time to obtain the desired dose at each time: 1 min for 31 L, 17 min for 537 L, and 30 min for 931 L. We have confirmed that the evaporation at RT preserves the integrity of the molecule, with no fragmentation, through Quadrupole mass spectrometry measurements.

Theoretical calculations were performed using Fireball DFT^29^ method with BLYP exchange–correlation functional^30^ and D3 corrections.^31^ We used a basis set of optimized numerical atomic-like orbitals (NAOs)^32^ with a 1s orbital for H, sp3 orbitals for C, N and sp3d5 orbitals for S and Mo atoms. The free energy profile was calculated using WHAM methodology, for each umbrella sampling window of WHAM we run an ab initio MD using Fireball DFT of 2000 steps at 300 K with a time step of 0.5 fs.^33^

## Results and Discussion

### Pristine MoS_2_ Single Crystal

Prior to any functionalization process, we investigated the 2H-MoS_2_ pristine surface by AFM and STM. Bulk MoS_2_ is a semiconductor with an indirect bandgap of 1–1.4 eV^34–36^ with lattice parameters of *a* = 3.16 Å and *c* = 12.29 Å.^37^ In terms of surface morphology, this material presents a flat surface with large terraces and low roughness. Figure 1a shows a representative AFM image of an area of 5 × 5 μm^2^ where a large terrace together with atomic step edges at the top of the image are visible. The density of intrinsic defects in pristine MoS_2_, mainly sulfur vacancies, depends on the synthesis method used for crystal growth. Synthetic single crystals present a low density of defects compared to natural MoS_2_ single crystals. No defects are observed when measuring with AFM due to the limitation in the lateral resolution of this technique. A detailed study of the height distribution (inset Figure 1a) reveals the flatness of the surface, with 1 Å of RMS roughness in the terraces, discarding step edges. The Gaussian fitting curve is centered on a 2.9 Å, with a full width at half maximum (fwhm) of 1.7 Å. To gain more insight on the morphology of the surface, AFM measurements were complemented with STM characterization. Figure 1b shows a representative STM image of an area of 100 × 100 nm^2^ (*I*_*t*_ = 0.4 nA and *V*_bias_ = 1.2 V) where randomly distributed intrinsic defects are visible, in good agreement with previous reported works.^38–40^ The shape of these defects depends on the STM measurement conditions and varies with the applied bias voltage.^41^ For positive bias voltages, the appearance of these defects is shown in Figure 1b. We have acquired and analyzed several images from different areas of the sample to increase the statistics on the density, shape, and size of the intrinsic defects and compare them with previous studies. This allowed us to determine whether the defects are single or double sulfur vacancies or even larger defects.^42^ The results are shown in Figure 1c,d. Most of the defects have a width (Figure 1c) ranging from 2 to 9 nm, although defects with widths exceeding 30 nm have been occasionally observed. Compared with the literature, where width values ranging from 3 to 5 nm have been assigned to single vacancies,^38^ we can identify a fraction of approximately 33% of the total intrinsic defects as single sulfur vacancies (highlighted in Figure 1c). We have determined that the intrinsic defect density is of 6 × 10^10^ defects/cm^2^, implying a surface density ranging from 0.6% to 2%. Previously reported values range from 1.5% to 10%.^38^ In contrast, most defects have depths of less than 1 Å (Figure 1d), much smaller than those reported by Addou and co-workers, where values of 5 Å were found.^38^ The discrepancy in depth values stems from the different bias values used for image acquisition. We obtained depths of 1 Å with a bias greater than +1 V (+1.2 V in Figure 1 b), while reported values of around 5 Å were obtained with a bias of −0.3 V.^38^ A higher density of vacancies can be achieved through controlled Ar^+^ sputtering of the surface, however the sample also becomes rougher after this type of treatment.

**Figure 1:**
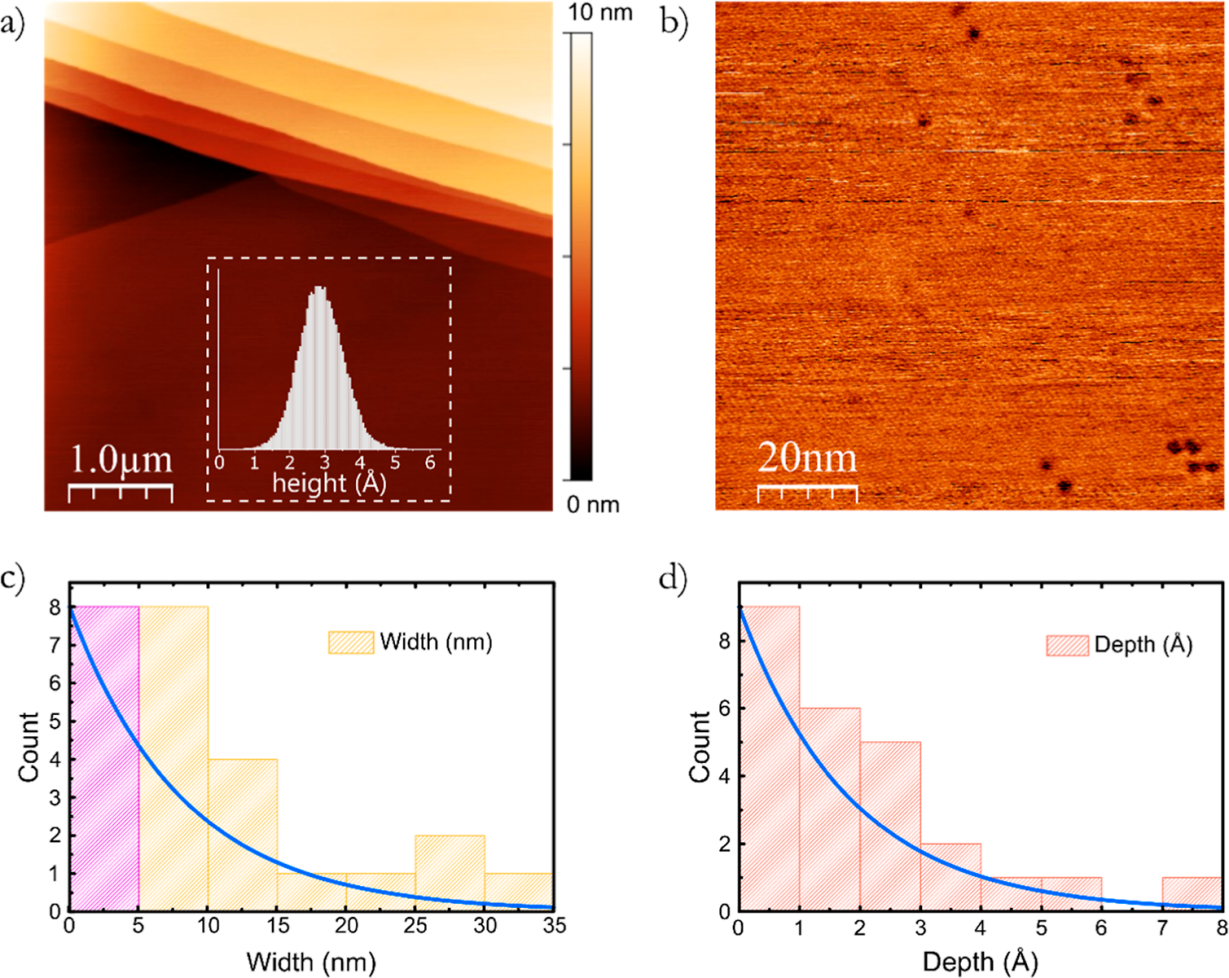
Morphological characterization of the pristine MoS_2_ surface. (a) AFM image of 5 × 5 μm^2^ where a large terrace is visible together with atomic step edges on top of the image. The inset shows the height distribution. (b) STM image of a 100 × 100 nm^2^ area in a terrace (*I*_*t*_ = 0.4 nA, *V*_bias_ = +1.2 V) where defects as dark features are visible. Distribution of (c) widths (the purple bar highlights single vacancies) and (d) depths of the intrinsic defects on MoS_2_ surface. The blue line is drawn as a guide to the eye.

### Functionalization with 4-Aminothiophenol

4-ATP molecules (sketch in the inset of Figure 2a) were deposited on the surface through direct evaporation in UHV at different doses (31 L, 537 and 931 L). The high diffusion of molecules on the surface is evident in the STM images (Figure S1) where the molecules can barely be seen because they diffuse faster than the scanning speed. We inspected the surface after evaporation of each dose by acquiring AFM images of different areas in ambient conditions. Figure 2a,b show the molecules on the surface after evaporating 31 and 537 L, respectively. In both images, the molecules appear as bright protrusions. The height of the protrusions observed in Figure 2a is 2–2.5 nm which may imply that instead of independent molecules, small clusters of molecules are formed and pinned in the step edges, this implies a low adsorption energy that facilitates high molecular diffusivity, leading to preferential aggregation at step edges, which represent the most chemically reactive sites on the surface. The height distribution of the molecules shown in Figure 2b will be thoroughly studied in the next section. Finally, we achieved near-complete surface coverage by exposing the sample to a dose of 931 L with a height of 0.4 nm (Figure 2c). The molecular density increases with the dose. A minimum density of 2 × 10^10^ molecules/cm^2^ was obtained for 31 L, while for 537 L a minimum density of 1.1 × 10^12^ molecules/cm^2^ was obtained (densities were obtained from statistics of several images). These values are close to those reported for molecules in artificially generated vacancies in graphene through Ar^+^ sputtering, where the values are of the order of 10^12^ molecules/cm^2^.^7^ Since the density of the molecules for a dose of 537 L is higher than the density of the intrinsic vacancies, we cannot discard that a fraction of the molecules can be physisorbed on the surface after the vacancies have been saturated. To clarify this point, we increased the density of the vacancies by artificially generating additional vacancies through Ar^+^ sputtering in UHV until we reached a density of defects of the order of 1 × 10^14^ defects/cm^2^ and performed the same experiments, as it will be shown in the discussion. In the case of 931 L, we monitored a given area for several hours and observed the diffusion of molecules across the surface (Figure S2). The diffusion of molecules might indicate a weak interaction with the surface, i.e., a noncovalent bond between the molecule and the surface. In addition, we obtained large area STM images for 33 and 100 L, where nonsaturated defect sites were observed, even at the highest dosage, endorsing that defect sites do not act as reactive center for covalent functionalization (see Figure S3). For a deeper study of the functionalization degree of the MoS_2_ surface we have focused on the surface with the intermediate coverage (537 L).

**Figure 2:**
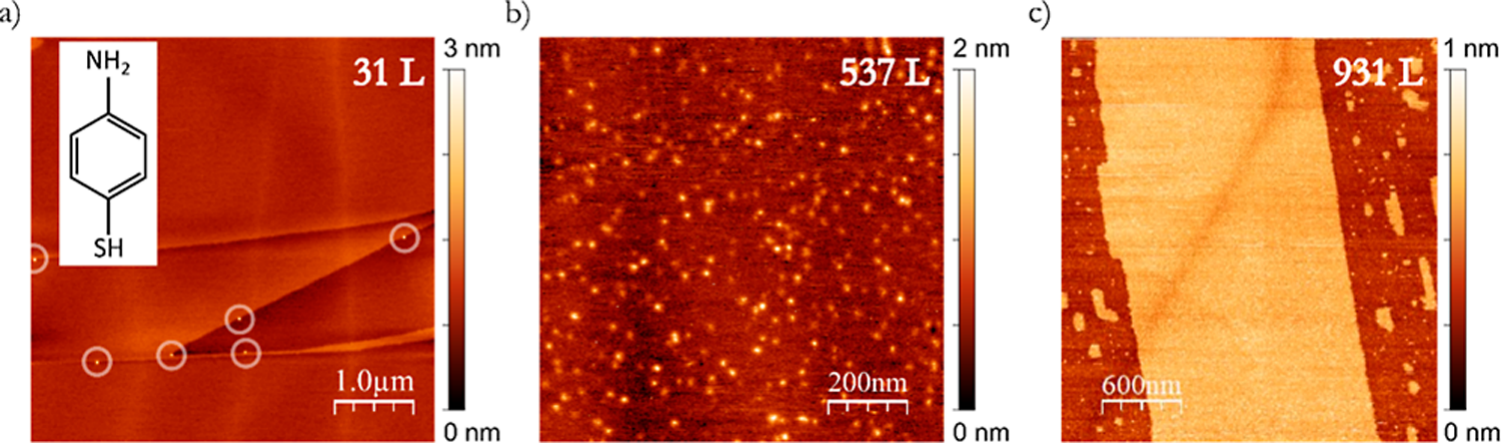
AFM images of the surface of MoS_2_ exposed to different doses of 4-ATP. (a) 5 × 5 μm^2^ image of the surface after an exposure of 31 L. Molecules appear as bright protrusions, highlighted by the white circles. The inset shows the sketch of the 4-ATP molecule. (b) 1 × 1 μm^2^ image of the surface after an exposure of 537 L. (c) 3 × 3 μm^2^ image of the surface after an exposure of 931 L.

It is important to note that diffusion was always present when measuring with both STM and AFM techniques. This behavior was evidenced by consecutive AFM images (Figure S2), where molecular positions changed over time, and by STM images (Figure S1), in which the molecules were difficult to image reproducibly because they diffused faster than the scanning velocity. Notably, this behavior was observed even at 77 K, the temperature at which the STM measurements were performed. We believe that these observations constitute strong evidence of high surface mobility, consistent with a low diffusion barrier and characteristic of physisorbed species rather than covalently bounded molecules.

As detailed in the experimental methods section, AFM measurements were performed in dynamic mode, using three different types of tips: one decorated with gold nanoparticles (AuNPs), an ultrasharp silicon tip, and a regular silicon tip. The following section presents the topographic analysis as a function of the tip and its interaction with the molecules on the surface. The height distributions are collected in Figure 3 and their representative AFM images shown in Figure S4. Figure 3a,b show the heights obtained by scanning molecules on a pristine MoS_2_ surface, with intrinsic vacancies, while Figure 3c and d show the heights obtained on a MoS_2_ surface with additional vacancies generated through Ar^+^ sputtering (additional AFM images of molecules on irradiated surfaces for different doses are shown in Figure S5). Figure 3a shows the height distribution of the protrusions observed in a given area of the sample scanned with an ultrasharp silicon tip, represented with green bars. The height distribution shows a median value of 3.3 Å with an interquartile range (IQR; Q1-Q3) of 2.7 Å—4.5 Å. Differences in heights from one run to another can be assigned to the evolution of the tip. The main point, however, is that all the observed molecules appear higher than the MoS_2_ surface for all cases when using a silicon tip. Figure 3b shows the results from the very same system as shown in Figure 3a with the single difference being that, in this case, measurements have been performed using a tip decorated with AuNPs instead of a silicon tip. Both distributions have been collected from the same system in the same experimental run. When measuring, the first scanned image present bright features (Figure S4b) and the median of the height distribution is 2.5 Å, with an IQR of 2.5 Å −3.5 Å. For the subsequent images, surprisingly the contrast has flipped, and we observe dark features (Figure S4b) with a median of the height distribution of −4.4 Å, with an IQR of −4.4 Å—–3.6 Å. We ascribe the increased dispersion of the height distribution in negative heights with respect to the positive ones to the additional variability in the measured signal produced by the functionalization of the AFM tip.

**Figure 3:**
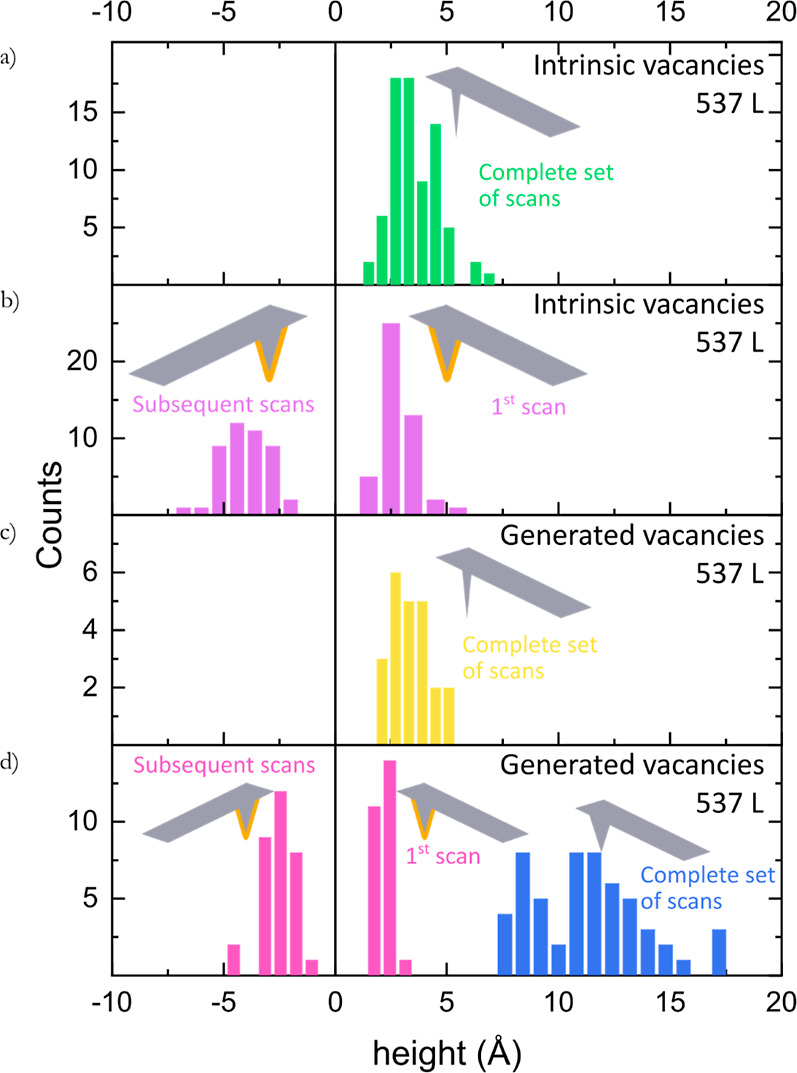
Histograms showing the height distribution of the molecules on MoS_2_ after an exposure of 537 L of 4-ATP, collected from AFM images acquired with different probes: (a) an ultrasharp silicon tip (pristine MoS_2_). (b) A AuNPs-decorated tip. Positive heights flip into negative heights after first image acquisition of molecules on pristine MoS_2_. (c) An ultrasharp silicon tip where height values are represented with yellow bars, for molecules on MoS_2_ with generated vacancies. (d) A silicon tip (blue bars) and a AuNPs-decorated tip (pink bars). Positive heights flip into negative heights after first image acquisition when measuring with AuNPs-decorated tip. Heights remain positive for all the measurements when using a silicon tip for molecules on MoS_2_ with generated vacancies.

This analysis has been completed with molecules deposited on surfaces with artificially generated vacancies by a controlled Ar^+^ bombardment of the surface (vacancy generation parameters: angle of incidence: 45° with respect to the normal of the surface; 15 s; *E*_ion_ = 250 eV; *I* = 0.7 μA; *P* = 7 × 10^–8^ mbar). These additional defects haven been made on UHV, ensuring that the vacancies are not saturated with contaminants (AFM images on Figure S5). Artificially generated vacancies may also not consist only of single vacancies, but additional divacancies or Mo–S vacancies may have been created. Figure 3c shows the height distribution of molecules on a MoS_2_ surface with generated vacancies, measured again with an ultrasharp silicon tip (Figure S4a). The median of the height distribution is 3.3 Å with an IQR of 2.7 Å–3.9 Å. We obtain the same results as in the case of the pristine sample with the same probe and no change in the contrast is observed. Finally, Figure 3d shows the height distribution of molecules on a surface with artificially generated vacancies, from images acquired with both a regular silicon tip and an AuNPs-decorated tip. The median of the height distribution, obtained with the silicon tip, is 11.6 Å, with a IQR of 9.2 Å–13.2 Å. When measuring with AuNPs-decorated tip, we observe again the inversion in the contrast. The first image shows positive height values while the subsequent ones show negative values with respect to the surface (Figure S4b). The median of the positive height distribution is 2.45 Å, with an IQR of 1.75 Å—2.45 Å. The median of the negative distribution is of −2.45 Å, with an IQR of −3.15 Å—–1.75 Å. We ascribe this singular change in the contrast to a modification of the probe as a result of the interaction of the gold tip with the thiol moieties of the molecules on the surface in both cases, with intrinsic vacancies and with additional vacancies, pointing out again to a weak interaction of the 4-ATP molecules with the MoS_2_ surface. If the molecules were covalently bonded to the defect sites, we would expect that the covalent bonding would impede the attachment to the probing tip and we would not see a contrast inversion when measuring with an AuNPs-decorated tip.

The contrast change of the features assigned to the molecules in the AFM topographic images depending on the probe material is discussed below, and we interpret it as a signature of the nonspecific, physisorption-like interaction of the molecules with the MoS_2_ surface.

In AFM, topographic images are obtained by keeping a selected tip–sample interaction constant through feedback control. In dynamic mode, as the tip interacts with the sample, the oscillation amplitude decreases, the resonance frequency can shift, and the phase can deviate from its free-air value. Any of these parameters can be used as the feedback signal, with the tip–sample distance continuously adjusted to keep it at the chosen set point.^43^ In our study, the amplitude is the chosen magnitude, and under our experimental conditions (nonmagnetic tips, ambient conditions), the interaction is dominated by van der Waals forces, which are distance-dependent and therefore directly correlate with the surface topography.

However, the assumption of a purely distance-dependent interaction does not always hold. The interaction can also vary with the local chemical nature of the surface regions. This cross-dependence may lead to apparent height artifacts in the reconstructed topography and even contrast inversion in the AFM images. This effect has been previously reported in systems including block copolymers, nanoparticles, and self-assembled monolayers on Au and mica.^44–47^

We therefore attribute the contrast inversion observed only when using AuNP-functionalized tips to a change in tip–sample interaction during scanning. Given the strong affinity of thiols for gold surfaces,^48^ if the 4-ATP molecules are not strongly bounded to MoS_2_, it is plausible that they detach from the substrate and attach to the Au-coated tip during the first scans (note this does not occur with Si tips). This implies, at the same time, that the thiol group has not undergone dehydrogenation and posterior healing of the sulfur vacancy. Once adsorbed onto the tip, the interaction during scanning is no longer governed by the Au-surface interaction, but rather by the amino group of 4-ATP (–NH_2_, inset of Figure 2a) interacting with the sample. This would account for the behavior observed in Figure 3b, where bright protrusions are visible in the first acquired image, while subsequent scans reveal depressions at the same locations.

Importantly, a change in tip–sample interaction does not automatically result in contrast inversion. For inversion to occur in our case, the 4-ATP-functionalized tip must interact more strongly and over a longer range with the MoS_2_ substrate than with the adsorbed 4-ATP molecules themselves. As a result, the feedback loop drives the tip closer to the molecule-covered regions to maintain the set point, which is interpreted as lower height in the topographic reconstruction.

The fact that this effect is only observed with AuNPs-decorated probes can be explained by the lower affinity of thiols for silicon surfaces, which prevents significant molecule attachment to bare Si tips.

To verify whether the contrast inversion is driven specifically by the thiol-gold affinity, we performed control experiments using 4-aminophenol-decorated surfaces imaged with AuNP-functionalized probes. As shown in Figure S6, the contrast inversion observed with 4-ATP does not occur in this system. This absence of inversion confirms that the hydroxyl group does not establish a sufficiently strong interaction with the gold tip to induce the same probe-mediated contrast changes, thereby isolating the Au–SH interaction as the primary mechanism behind the phenomena reported.

In addition, before measuring, amplitude–distance curves were acquired to ensure that comparable and low-interaction conditions were employed for all samples and tip types. Figure S7 shows a representative curve for the imaging conditions employed in this work, with a reduction factor *r* ≅ 0.9 (where *r* = *a*_set_/*a*_free_, i.e., the ratio between the set point amplitude and the free amplitude) and a tip–sample distance of approximately 10 nm. Amplitude values are reported in volts, as they directly reflect the photodiode output signal. As discussed in detail elsewhere,^49^ in amplitude modulation AFM the interaction is predominantly attractive at low interaction strength, whereas stronger interaction conditions may lead to intermittent or fully repulsive regimes. The parameters used in our measurements fall within the so-called attractive regime.

Previous studies addressing the calculation of energy dissipation associated with tip–surface interactions in tapping-mode AFM estimated that, for cantilevers and imaging conditions similar to those employed here, the maximum dissipated power ranges between 0.3 and 0.8 pW, corresponding to an energy dissipation of approximately 20–60 eV per oscillation cycle. This energy is dissipated over the entire tip–sample contact area, which spans several nanometers and therefore involves many atoms or molecules. Consequently, the effective energy transferred to an individual molecule is expected to be only a fraction of an electronvolt.^50,51^ For comparison, the dissociation energy of a Mo–S bond has been estimated to be approximately 3.5–4.0 eV per bond,^52^ substantially larger than the effective energy that can be delivered to a single molecule under our imaging conditions.

Our hypothesis differs from what has been reported so far, where the role of defects as reactive sites for thiol dehydrogenation and effective covalent functionalization has been accepted.^5,21,22^ In this case, no covalent functionalization occurs, but simple physisorption of the molecules to the surface, since the molecules attach easily to the gold tip.

To gain insight into the interpretation of the contrast inversion mechanism and support our hypothesis based on the detachment and further pinning of the molecule on the tip due to a noncovalent functionalization, we have calculated the energy profile and the free energy barriers of the thiol dehydrogenation pathway and performed simulations of the interaction between a gold AFM tip and the MoS_2_ surface, comparing the cases with and without 4-ATP molecules bound to the tip. The simulations enable us to assess the changes in both the strength and range of the tip–sample interaction.

Prior to that, the different adsorption geometries of 4-ATP on MoS_2_ were studied, concluding that the most plausible is the planar one, contrary to other studies with thiol-containing molecules that suggest an upright configuration.^22,23,53^ We have compared the energies of different adsorption geometries of the molecule on a pristine surface and on a surface with a surrounding sulfur vacancy, finding that in both cases the most stable geometry is the planar one (Figure 4a). There is a difference of 0.91 eV in the adsorption energy for the planar geometry with respect to the upright one with the thiol group pointing downward on a pristine surface, and of 0.65 eV on a defected surface. Figure 4a presents the molecule adsorbed on a pristine MoS_2_ surface (upper panel) and on a surface with a sulfur vacancy (lower panel), with different geometries. All the relative and absolute adsorption energies, together with the absolute interaction energies for the different adsorption geometries of the molecule on a pristine and defected surface are collected in Table ST1 in the Supporting Information. The relative adsorption energies (geometries 2–5) are referenced to the geometry that has the lowest adsorption energy (geometry 1): In geometry 1 the molecule is adsorbed in a planar configuration, with the hydrogen atom of the thiol moiety pointing toward the surface. In geometry 2 and 3 the molecule is also in a planar configuration but with the hydrogen atom of the thiol group pointing downward and upward, respectively. In geometries 4 and 5 the molecule adopts an upright position being the least energetically favorable. The relative and absolute values of the adsorption energies, together with the absolute interaction energies of the molecule on a pristine and a defected surface are collected in the table shown in Figure 4a.

**Figure 4:**
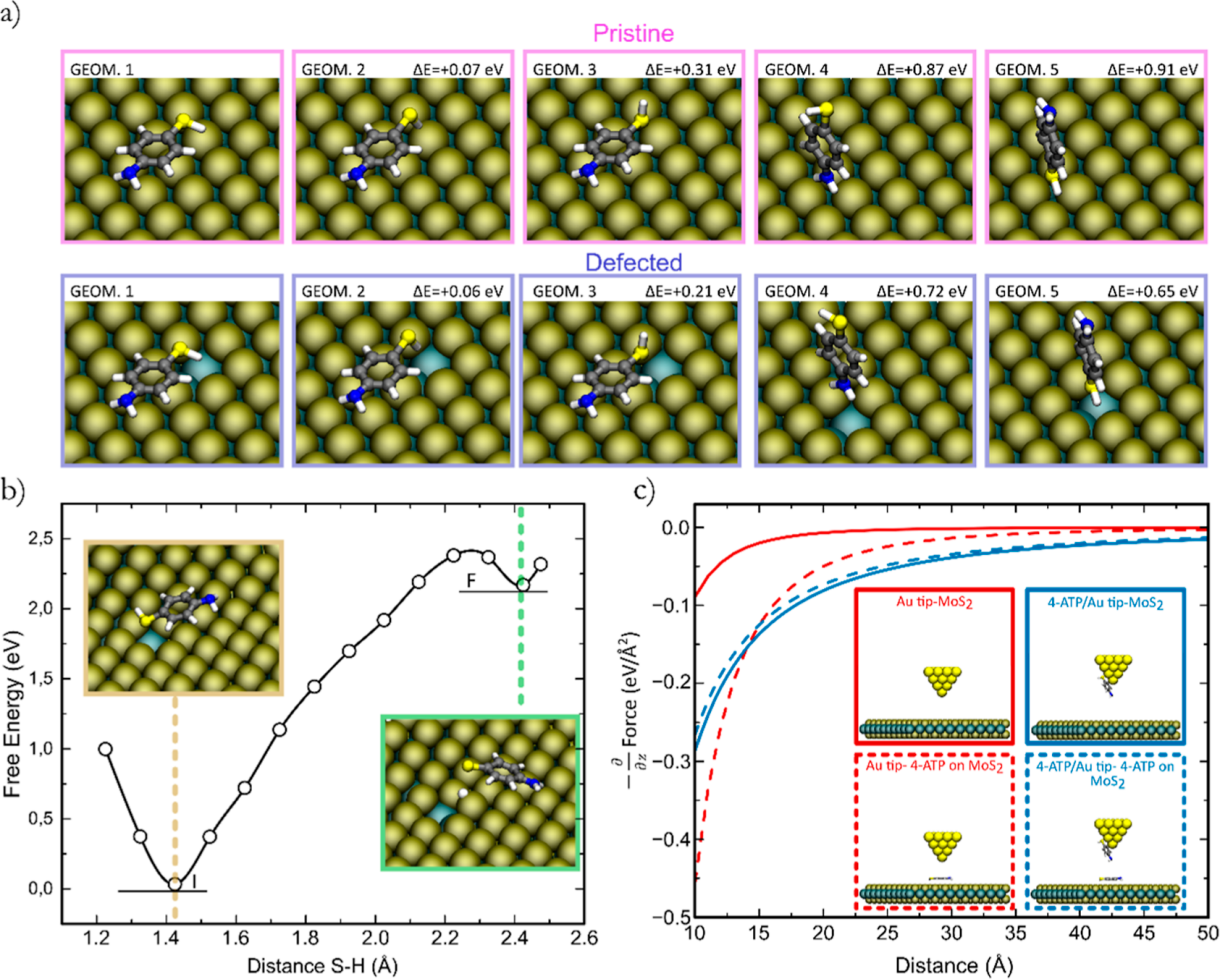
(a) Adsorption geometries (Geom. 1–5) of 4-ATP on MoS_2_ and their relative energies with respect to the lowest adsorption energy (Geom. 1). Upper panel: molecule on a pristine MoS_2_ surface. Lower panel: molecule on a defected MoS_2_ surface. (b) Energy profile and free energy barriers of the initial, intermediate, and final states of the reaction pathway for the dehydrogenation of the thiol moiety onto a surface with a sulfur vacancy. Inset: geometries of the initial (brown box) and final (green box) states. (c) Theoretical simulations of the derivative of the force vs distance curves of an AFM gold tip scanning a MoS_2_ surface for different experimental conditions. Red curves represent the gold AFM tip interacting with the bare surface (solid line) and with a functionalized surface (dashed line). Blue curves represent the gold AFM tip functionalized with a 4-ATP molecule, interacting with the bare surface (solid line) and with a functionalized surface (dashed line). Inset: sketches of the tip–sample conditions.

For a thiol to become covalently bonded to a sulfur vacancy, a prior step involving the dehydrogenation of the thiol is required. It has been previously reported that, in some cases, thiol-containing molecules can interact with sulfur vacancies through a quasi-covalent interaction in the absence of dehydrogenation. However, these studies also indicate that the strongest and most stable interactions correspond to fully covalent bonding, which typically occurs only after dehydrogenation of the thiol group.^54^ In order to demonstrate that the dehydrogenation does not take place in this case, we have calculated the energy profile and the free energy barriers for the reaction pathway of the thiol dehydrogenation in a sulfur vacancy. Figure 4b shows the energy profile together with the geometry of the initial (brown box inset) and final (green box inset) states. The intermediate states of the energy profile are shown in Figure S8. According to our findings, the system has to overcome an energy barrier of 2.5 eV to dehydrogenate. The free energy barrier values presented in other works are 0.48–0.51 eV when interacting with a sulfur vacancy on a MoS_2_ surface.^26,55^ The difference between the energy barrier we obtain and the values reported in the literature originates from both the specific molecule employed in each case and the geometrical configuration of the molecule on the surface. Due to the strong influence that molecular orientation on the surface has, we have carefully calculated the adsorption energy differences for each possible molecular configuration (Figure 4a). Our calculations show that the most stable adsorption geometry for this molecule is the planar configuration (Figure 4a, geometry 1).

However, to decrease the energy barrier for a dehydrogenation event mediated by a sulfur vacancy, the molecule must reorient into an upright configuration, with the thiol group pointing toward the vacancy site—a process that itself entails an energy cost (Figure 4a, geometry 5).

At room temperature, this barrier is so large that the system cannot effectively overcome it, while such a process may indeed occur at elevated temperatures (exceeding 700 °C). This thermal regime would inevitably induce morphological degradation of the MoS_2_ lattice. The concomitant generation of sulfur vacancies and other point defects would fundamentally alter the sample stoichiometry, thereby compromising the original experimental parameters.^56^

Finally, and to explain the origin of the contrast inversion found during the image acquisition with the AFM, we have calculated the derivative of the force vs distance curves for four different tip–surface conditions: (i) a gold AFM tip interacting with the bare MoS_2_ surface, (ii) a gold AFM tip interacting with 4-ATP molecules on the MoS_2_ surface, (iii) a functionalized gold AFM tip with a 4-ATP molecule that is attached to the probe through the thiol group of the molecule and thus interacting with a pristine MoS_2_ surface via the NH_2_ group, and (iv) the same functionalized gold AFM tip interacting with a functionalized MoS_2_ surface. We have calculated the relative adsorption energies depending on the orientation of the molecule attached to the tip, finding the thiol-gold attachment the most energetically favorable (see Figure S9). The curves have been fitted to an exponential function (see Figure S10). The derivative of the force vs distance has been calculated as the derivative of the exponential curve, that is, the interaction of the AFM tip as a function of the tip–surface distance, since the amplitude of the oscillation of the AFM cantilever is proportional to the derivative of the force between tip and sample.^57^ The $-\frac{\partial F}{\partial z}$ vs distance curves are shown in Figure 4c, where the inset presents the tip–sample conditions for each curve. The red curves correspond to a gold AFM tip interacting with the bare MoS_2_ surface (solid red curve) and to the same gold AFM tip interacting with 4-ATP molecules on the MoS_2_ surface (dashed red curve). The blue curves correspond to the gold AFM tip with a 4-ATP molecule attached. The solid blue curve corresponds to the interaction between this tip and the bare MoS_2_ surface, whereas the dashed blue curve represents the interaction with the molecules on the surface. We have ranged the simulation to the distances where the effective interaction between the tip and the surface is relevant, which in the case of AFM is within 1 and 5 nm. In the case of the gold AFM tip (red curves), the interaction, at a given distance, is greater for the dashed curve than for the solid curve, which implies that the molecule appears brighter than the surface in image acquisition. Interestingly, in the case of the blue curves, the solid curve presents a stronger interaction than the dashed one, entailing that the surface appears brighter than the molecules. The stronger long-range interaction for the molecule-functionalized tip (solid blue curve) causes the feedback loop to push the tip closer to molecule-covered areas, registering them as depressions (lower apparent height) in the topographic image. This means that the molecules will appear as depressions or dark features as demonstrated with the AFM experimental images (Figures 3 and S4).

These theoretical results reinforce the idea that the contrast inversion shown experimentally is due to the weak interaction of the 4-ATP molecules with the MoS_2_ substrate, which easily stick to the tip, producing a drastic change in the tip–surface interaction.

This noncovalent functionalization is independent of the number of sulfur vacancies, as opposed to what has been previously reported in literature.

## Conclusions

We have studied the interaction of 4-ATP molecules with the surface of MoS_2_ single crystals. We demonstrate how a controlled evaporation in an UHV environment and at RT provides a total control over the purity of the evaporated molecules, avoiding any type of solvents or residues and thus offering the possibility of studying the behavior of the adsorbed molecules for different doses evaluating the nature of the molecule–surface interaction.

We show that, under these conditions, covalent conjugation of sulfur with molybdenum atoms does not occur in the vacancies since the thiol group does not undergo dehydrogenation. Consequently, the molecules are weakly adsorbed onto a planar geometry, allowing them to diffuse across the surface and even attach to an Au probe at the AFM. The free energy barrier required for dehydrogenation is very high to be overcome at RT, and the contrast inversion images provided by the AFM experiments demonstrate our findings. Our study indicates that covalent functionalization of MoS_2_ by thiolated molecules is not straightforward, and that the role of sulfur vacancies is less significant than previously suggested.

## Supplementary Material



## Data Availability

Data will be made available on reasonable request.

## Notes

Supporting Information STM characterization of 4-ATP molecules on MoS_2_. AFM movie frames of 931L of 4-ATP on MoS2. Large area STM images of 4-ATP molecules on MoS_2_ surface. AFM images acquired with silicon and AuNPs-decorated tips. 4-ATP molecules on irradiated MoS_2_ surfaces. AFM control experiments with 4-aminophenol molecules. Representative curve for AFM imaging conditions. Intermediate states of the dehydrogenation pathway of the thiol moiety. Different geometries of the 4-ATP molecule attached to the AFM tip. Simulated energy vs distance AFM curves. Calculated adsorption and interaction energies of 4-ATP on a pristine and a defected MoS_2_ surface (PDF)
