# Supplementary material for: Mechanistic Insights into MoS2 Functionalization via Thiol Groups of 4‑Aminothiophenol
Source: Langmuir. 2026 Jun 29;42(27):19589–98. doi: 10.1021/acs.langmuir.6c01436 (PMC13374378; doi:10.1021/acs.langmuir.6c01436)
Supplement: Supplementary file 1 [file la6c01436_si_001.pdf]

Supplementary information

Mechanistic insights into MoS_2 Functionalization via Thiol Groups of 4-Aminothiophenol

Jon Azpeitia[1], Jesús Ignacio Mendieta-Moreno[1], Laia León-Boigues[1], Mar García-Hernández[1], José Ángel Martín-Gago[1], Carmen Munuera[1]*, Irene Palacio[1]**

[1]Instituto de Ciencia de Materiales de Madrid, CSIC, Sor Juana Inés de la Cruz 3, Cantoblanco, 28049 Madrid, Spain.

*Corresponding authors: jon.azpeitia@icmm.csic.es, cmunuera@icmm.csic.es, i.palacio@csic.es

Table of Contents

1. **STM characterization of 4-ATP molecules on MoS_2**
2. **AFM movie frames of 931L of 4-ATP on MoS_2**
3. **Large area STM images of 4-ATP molecules on MoS_2 surface**
4. **AFM images acquired with silicon and AuNPs-decorated tips**
5. **4-ATP molecules on irradiated MoS_2 surfaces**
6. **AFM control experiments with 4-aminophenol molecules**
7. **Representative curve for AFM imaging conditions**
8. **Intermediate states of the dehydrogenation pathway of the thiol moiety**
9. **Different geometries of the 4-ATP molecule attached to the AFM tip**
10. **Simulated energy vs distance AFM curves**
11. **Calculated adsorption and interaction energies of 4-ATP on a pristine and a defected MoS_2 surface**

1. STM characterization of 4-ATP molecules on MoS_2

Measurements of STM at 77 K after the evaporation of 33L of 4-ATP molecule. Molecules appear as blurry bright protrusions due to the diffusion that present across the surface.

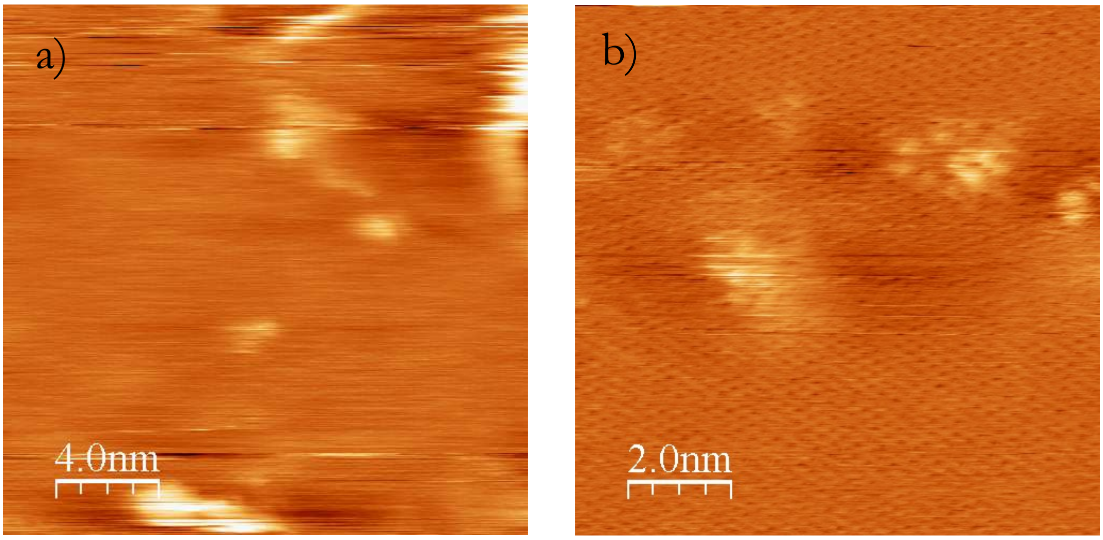

Figure S1. STM images of molecules (33L) on MoS_2 of an area of a) 20x20 nm^2 and b) 10x10 nm^2. Measurement conditions: I_t= 100 pA and V_{bias}= -800mV.

2. AFM movie frames of 931L of 4-ATP on MoS_2

Figure S2 a)-d) shows AFM movie frames composed of images centered in a unique zone for hours. The mobility of the molecules on the surface suggests that the interaction with the surface is weak, discarding a covalent conjugation with the Mo atoms of the surface.

Images a) to c) were acquired consecutively, with the parameters above, and thus the total time elapsed between the first and the third image is 57 min. Image d) was acquired 7 hours after image c), with the same parameters as a)-c). During that time the scanning was stopped and the tip retracted from the surface. The structural modifications observed in the subsequent image (Figure S2 d), recorded after an interval of several hours, confirm that the observed phenomena are intrinsic to the system and not an artifact or a tip-induced effect.

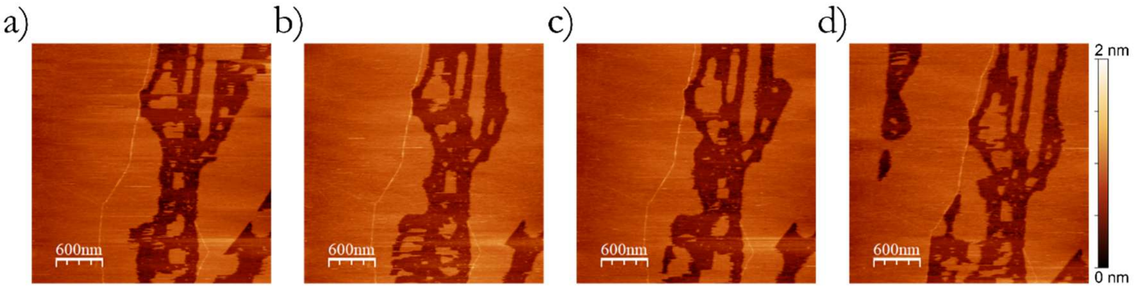

Figure S2. The figure corresponds to image frames (a-d) acquired at the same zone over the time. It evidences that molecules are not covalently bonded to the surface and diffuse across it. The color scale is the same for all the images.

3. Large area STM images of 4-ATP molecules on MoS_2 surface

Figure S3a and S3b show a pristine MoS_2 surface with intrinsic defects after the evaporation of 33 L and 100 L of 4-ATP, respectively. The images show unoccupied defect sites and blurry molecular features, together with stripe-like lines. Figure S3c shows an STM image of a defective MoS_2 surface after the evaporation of 33 L of 4-ATP. In the same image, the presence of empty defects, together with blurry molecular features and characteristic stripe-like lines, provides direct evidence of two key points. First, the blurred appearance and stripe patterns indicate that the molecules are diffusing across the surface, even at 77 K, demonstrating that they are not covalently bound to the substrate. Second, the observation of unoccupied vacancies clearly shows that the molecules do not preferentially bind to sulfur vacancies, but instead remain physisorbed on the surface.

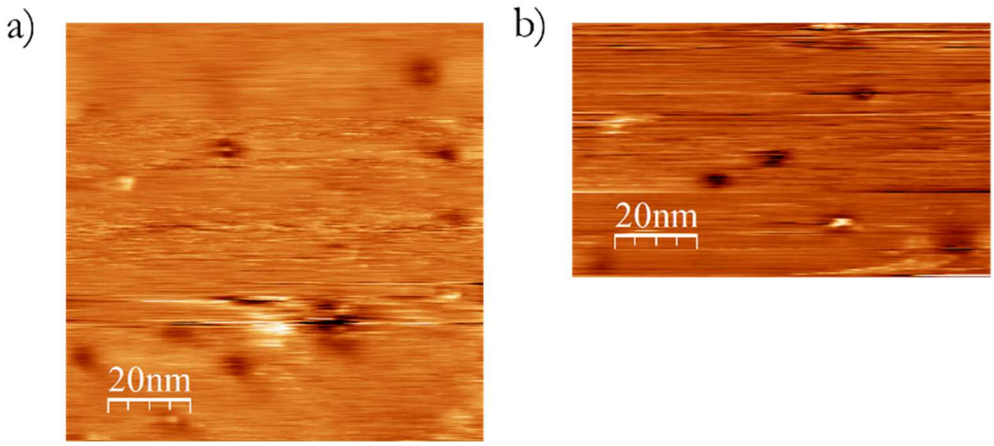

Figure S3. Large area images of the MoS_2 surface exposed to a) 33 L and b) 100 L of 4-ATP molecules.

4. AFM images acquired with silicon and AuNPs-decorated tips

Figure S5 shows the representative AFM images of 4-ATP molecules on MoS_2 acquired with silicon tips (Figure S5a) and with AuNPs-decorated tips (Figure S5b) for a dose of 537 L. Figure S4a shows bright protrusions in all cases: in the image that belongs to molecules on pristine MoS_2 with intrinsic vacancies (upper panel) that have been acquired with an ultra-sharp silicon tip, and in the images corresponding to molecules on MoS_2 with generated vacancies (lower panel) acquired with an ultra-sharp and a regular silicon tip. Figure S5b shows the contrast inversion when measuring with an AuNPs-decorated tip for both cases, molecules on a pristine MoS_2 surface with intrinsic vacancies (upper panel), as well as for molecules on MoS_2 with generated vacancies. While silicon tips always show the molecules as bright protrusions, AuNPs-decorated tips present a contrast inversion after the first image acquisition. Figure S5 shows representative AFM images from which the height distributions of Figure 3 in the main text have been obtained.

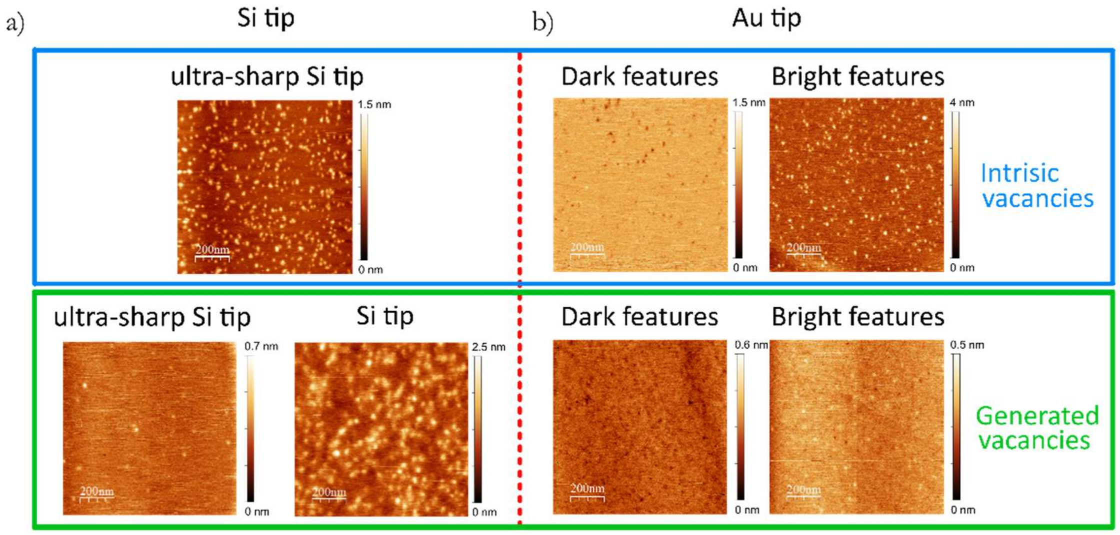

Figure S4. Representative AFM images acquired with a) silicon tips and b) AuNPs-decorated tips. Upper panel shows images of the molecules on a pristine MoS_2 surface, with intrinsic vacancies (blue box). Lower panel shows images of the molecules on a MoS_2 surface with generated vacancies (green box).

5. 4-ATP molecules on irradiated MoS_2 surfaces

Figure S4 shows AFM images at different evaporation doses of irradiated MoS_2 surfaces, acquired with an AuNPs tip. Figure S4a and b show a dose of 157 L and Figure S4 c and d a dose of 537 L. The surface has been previously irradiated with Ar^+ ions, with an ion dose of 5.7×10^{13} ions/cm^2. The ion dose was calculated from the following relation: $ion\ dose = \frac{I.t}{A.e}$ where I is the ion current, t is the time of irradiation, A is the area of the sample, and e is the charge of the electron. The roughness of the surface increases significantly. For both doses, the contrast inversion takes place when measuring with AFM when employing Au NPs tip. Bright features (Figure S4a and c) turn into dark features (Figures S4b and d).

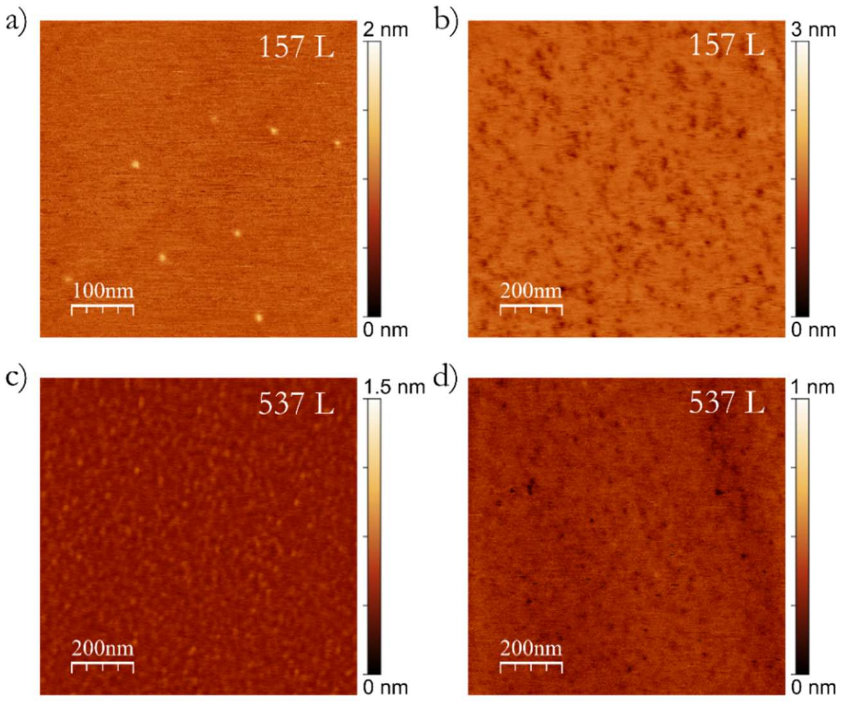

Figure S5. AFM images of 4-ATP molecules on irradiated zones of the surface, acquired with a AuNP tip. Doses of a) and b) 157 L and c) and d) 537 L.

6. AFM control experiments with the 4-aminophenol molecules

Figure S6 shows the control AFM experiment employing a not thiol-containing molecule (4-aminophenol). The sample was exposed to a dose of 537 L. We observe that after several images there is no contrast inversion due to the interaction between molecule and probe.

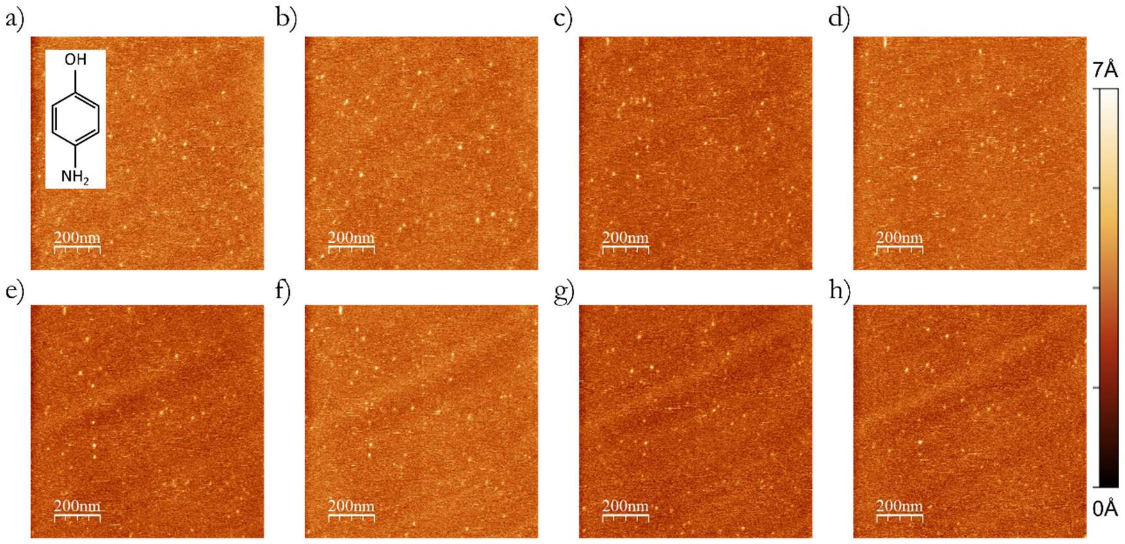

Figure S6. a)-h) Consecutive AFM images of 4-aminophenol on MoS_2. Inset of a) shows the sketch of the molecule.

7. Representative curve for AFM imaging conditions

Figure S7 shows the representative curve of the imaging conditions employed in AFM measurements. The parameters used in our measurements fall within the so-called attractive regime.

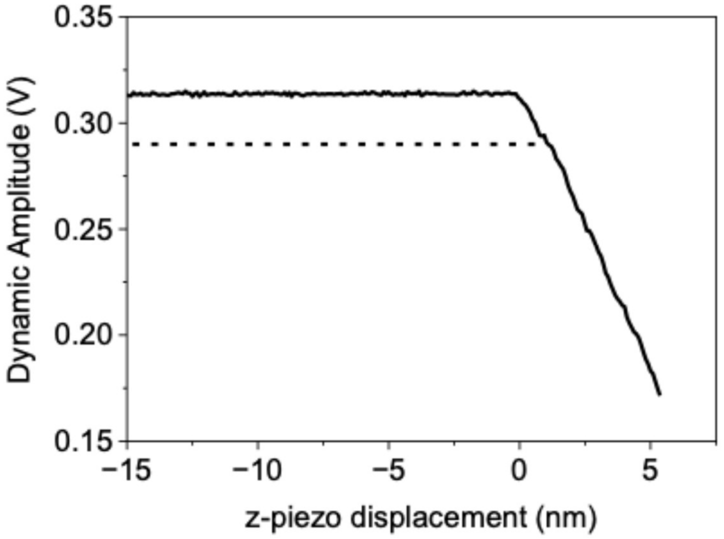

Figure S7. Amplitude–distance curves acquired with a silicon tip on the MoS_2 sample exposed to 537 L of 4-ATP. Dashed lines indicate the selected set-point value.

8. Intermediate states of the dehydrogenation pathway of the thiol moiety

Figure S8 shows the energy profile for dehydrogenation of a thiol on a defected MoS_2. The molecular geometries of the intermediate states are shown on top of the curve, with a color curve that can identify each position in the curve, together as increasing numbers as identifier labels.

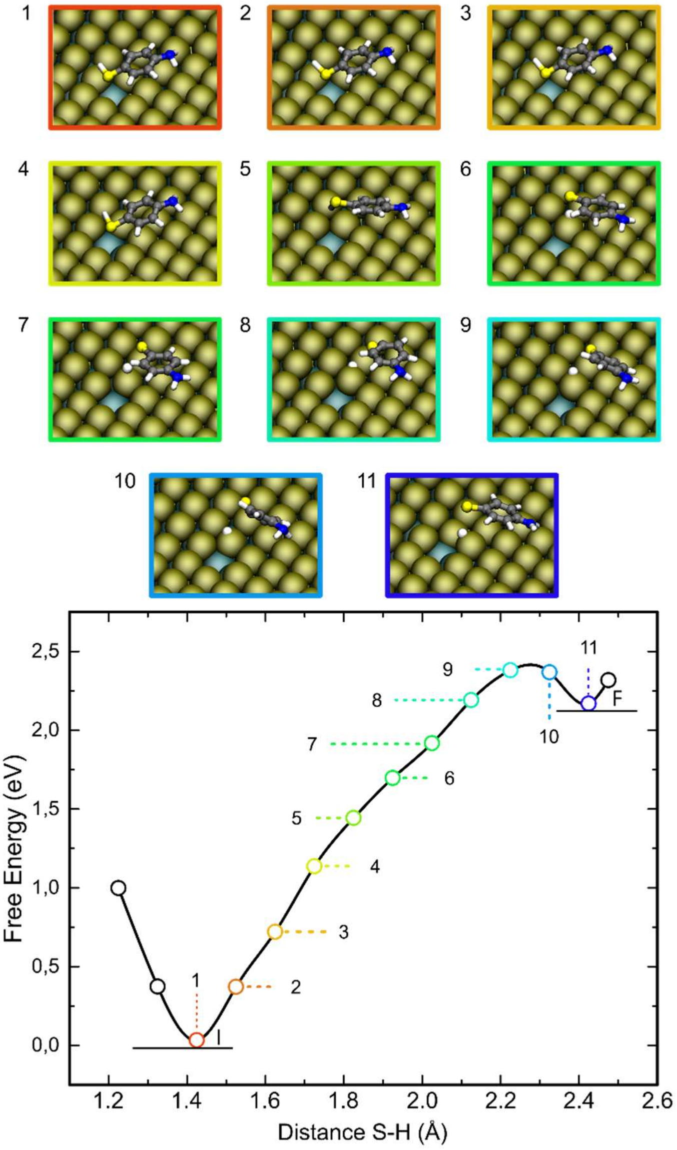

Figure S8. Intermediate states between the initial and final states (dehydrogenation of the thiol moiety) of the 4-ATP molecule on top of the surface in the vicinity of a sulfur vacancy.

9. Different geometries of the 4-ATP molecule attached to the AFM tip

Figure S9 shows the different calculated configurations of the molecule attached to the AFM tip, with the relative adsorption energies for each case. It is deduced that the most favorable configuration is when the molecule is linked through a thiol-gold interaction.

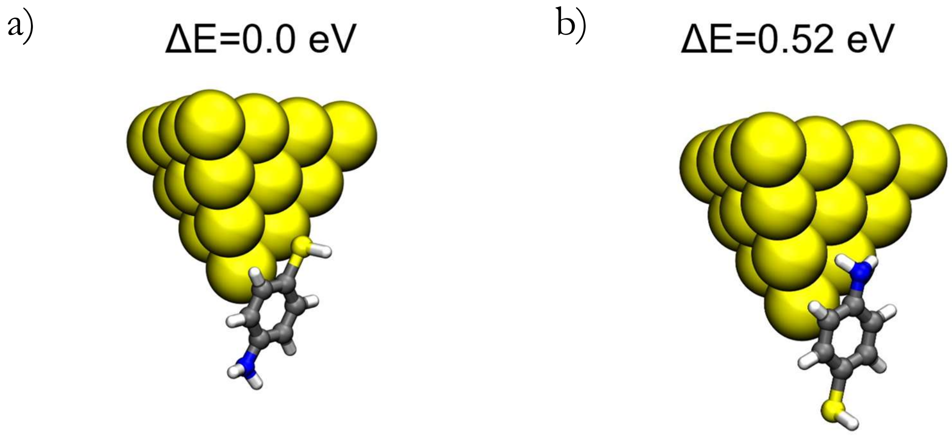

Figure S9. 4-ATP molecule attached to the AuNPs-decorated AFM tip from a) the thiol group and b) the amino group.

10. Simulated energy *vs* distance curves

We have calculated the energy *vs* distance curve using Fireball DFT method optimizing the structure for each height. The numerical data for each curve is fitted employing non-linear least squares to the function: $Ad^B + C$; where d is the distance between the surface and the tip and A, B and C are free parameters.

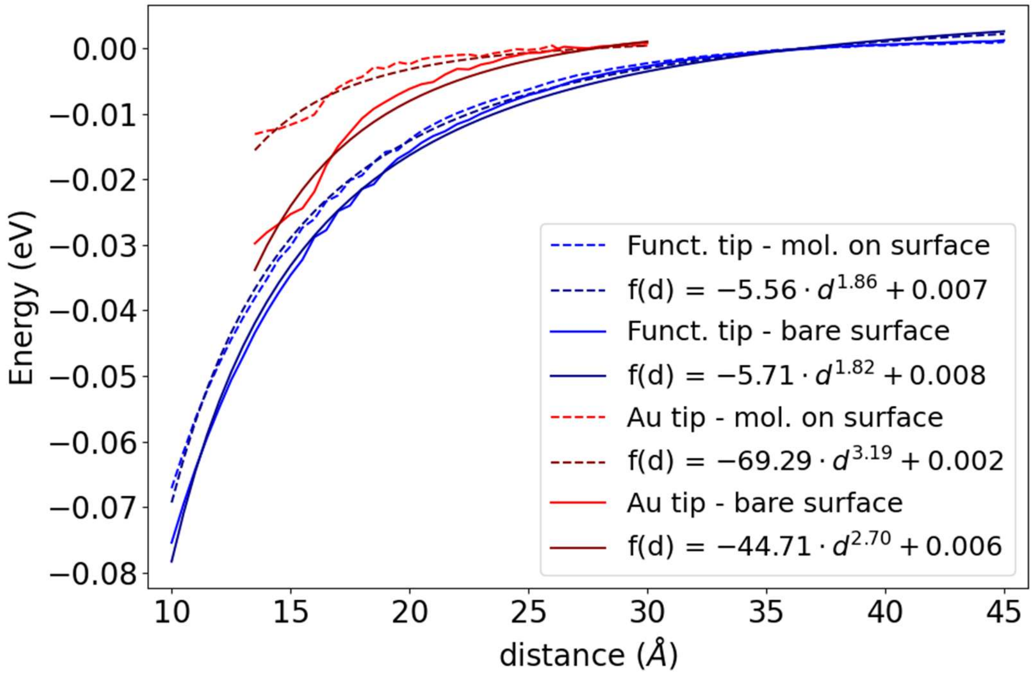

Figure S10. Curves of energy vs distance to the surface with the bare tip (red curves) and with the tip with a molecule attached (blue curves). The solid lines represent the interaction with the MoS_2 while the dashed lines represent the interaction with the 4-ATP molecules. The dark blue and dark red curves correspond to the fitting of the curves.

11. Calculated adsorption and interaction energies of 4-ATP on a pristine and a defected MoS_2 surface

	GEOM. 1	GEOM. 2	GEOM. 3	GEOM. 4	GEOM. 5
**$\Delta E_{ads\ (pristine)}$ (eV)**	0.0	0.07	0.31	0.87	0.91
**$\Delta E_{ads\ (defected)}$ (eV)**	0.0	0.06	0.21	0.72	0.65
**$E_{ads\ (pristine)}$ (eV)**	-1.22	-1.15	-0.91	-0.35	-0.31
**$E_{ads\ (defected)}$ (eV)**	-1.17	-1.11	-0.96	-0.45	-0.52
**$E_{int\ (pristine)}$ (eV)**	-1.38	-1.33	-1.15	-0.46	-0.33
**$E_{int\ (defected)}$ (eV)**	-1.31	-1.36	-1.20	--0.58	-0.44

Table ST1. Relative and absolute adsorption energies, and the absolute interaction energies for different geometries of the 4-ATP molecule on a pristine and a defected MoS_2 surface.
